# The relationship between family socioeconomic status and the young children’s number sense development: The mediating role of parent-child activities

**DOI:** 10.1371/journal.pone.0301758

**Published:** 2024-04-18

**Authors:** Ying Jiang, Xiaofen Wang

**Affiliations:** 1 Fujian Institute of Education, Fujian, China; 2 Chengyi College, Jimei University, Fujian, China; University of Rome La Sapienza: Universita degli Studi di Roma La Sapienza, ITALY

## Abstract

This study examines the relationship between family socioeconomic status, parent-child activities, and young children’s digital development. Using the Family SES questionnaire, the parent-child activities questionnaire, and the 5 to 6 year old children’s number sense development scale as research instruments, 314 young children (*M* = 70.42, *SD* = 3.47). The results show that: (1) Family Socioeconomic Status can significantly positively predict the young children’s number sense development; (2) Parent-child activities play a partial mediating role in the relationship between family socioeconomic status and the young children’s number sense development. This study provides specific guidelines and recommendations for improving the young children’s number sense development.

## Introduction

Number sense refers to the understanding of counting, numbers, calculations, and digital systems and structures [1]. It is considered as one of the basic mathematical literacies of human beings and the basis for establishing a clear concept of numbers, effectively carrying out mathematical activities such as calculation, and linking mathematics with practical problems [2]. Number sense plays an important role in people’s understanding of more complex algorithms and in gaining mathematical experience and skills [3, 4].

Studies have found that the number sense performance in young children’s kindergarten is significantly related to their math scores at the end of the third grade, and it is a strong predictor of math scores in primary school. Furthermore, the development level of number sense in early childhood can effectively predict the performance of standardized math tests after young children enter school and be a powerful predictor of young children’s future math difficulties [5, 6]. Therefore, it is of great practical significance to study the influencing factors and mechanism of young children’s number sense ability. This will aid in formulating strategies to cultivate young children’s number sense development and lay a foundation for their future mathematical development.

Research shows that the socioeconomic status (SES) of a family is an important variable that affects children’s academic achievement [7, 8]. Foreign studies indicate that children from low SES families are at a disadvantage in number sense performance compared to their peers with middle and high SES. They are also more likely to become a high-risk group for math difficulties in the future [9–11]. The development of children with low SES in counting, number relations, and number operations is delayed [12]. This difference has been observed before children enter kindergarten and persists even after kindergarten education, becoming even more pronounced [13]. After formal schooling, these low SES children continue to face serious academic problems in math [14, 15]. It is evident that family SES is an important variable that influences young children’s number sense development.

However, compared to the close attention paid by foreign countries to the relationship between SES and children’s number sense development, domestic research on the number sense development started late. Currently, the primary focus of domestic research is on school-age children, with research content primarily concentrating on the correlation between SES and the mathematics academic performance of school-age children [16, 17]. Little attention has been given to preschool children’s number sense development, and there is limited research on the correlation between SES and the young children’s number sense development, necessitating Chinese-based data to complement existing research in China.

Therefore, based on previous studies, this research will investigate the relationship between SES and the young children’s number sense development in China, and delve deeply into the mechanism through which family SES affects children’s number sense development. Additionally, this study assumes that family SES can significantly and positively predict the development level of children’s number sense (H1).

The study also points out that the SES of a family, as a background feature of a class group, has a wide influence on the family dynamic process and individual behavior [18, 19]. The SES of the family not only affects the individual development results but also affects the role of parents in the development of their children. Vygotsky pointed out that parent-child interaction experience is an important mechanism for children’s cognitive development [20, 21]. Family investment theory also suggests that effective interaction between parents and children is often more conducive to children’s cognitive development than the provision of family material resources [22, 23].

Therefore, in recent years, researchers have paid increasing attention to the influence of family parent-child activities on young children’s number sense development. Previous studies have shown that parent-child mathematical activities can impact the development of children’s mathematical abilities [24–26]. Good math parent-child activities can effectively promote the young children’s number sense development [27, 28].

However, previous studies have only examined the relationship between singular mathematics-related parent-child activities and number sense development. In reality, parent-child interaction within families is varied and comprehensive, encompassing various aspects, rather than solely focused on mathematical experiences. Furthermore, children’s early number sense development is influenced by numerous factors. Research on children’s early experiences in digital cognitive development and language development demonstrates that language factors play a crucial role in shaping digital concepts [29]. The lack of language comprehension may impede problem-solving and affect the expression of mathematical concepts and understanding computational concepts [30, 31]. Research has shown that quantitative and spatial abilities (e.g., building blocks, picture completion) are positively correlated [32–34]. Based on this, it can be inferred that in addition to math parent-child activities, children’s experiences gained from other parent-child activities, such as language experience from parent-child reading and spatial ability from parent-child building block activities, will also affect young children’s number sense development.

**Fig 1:**
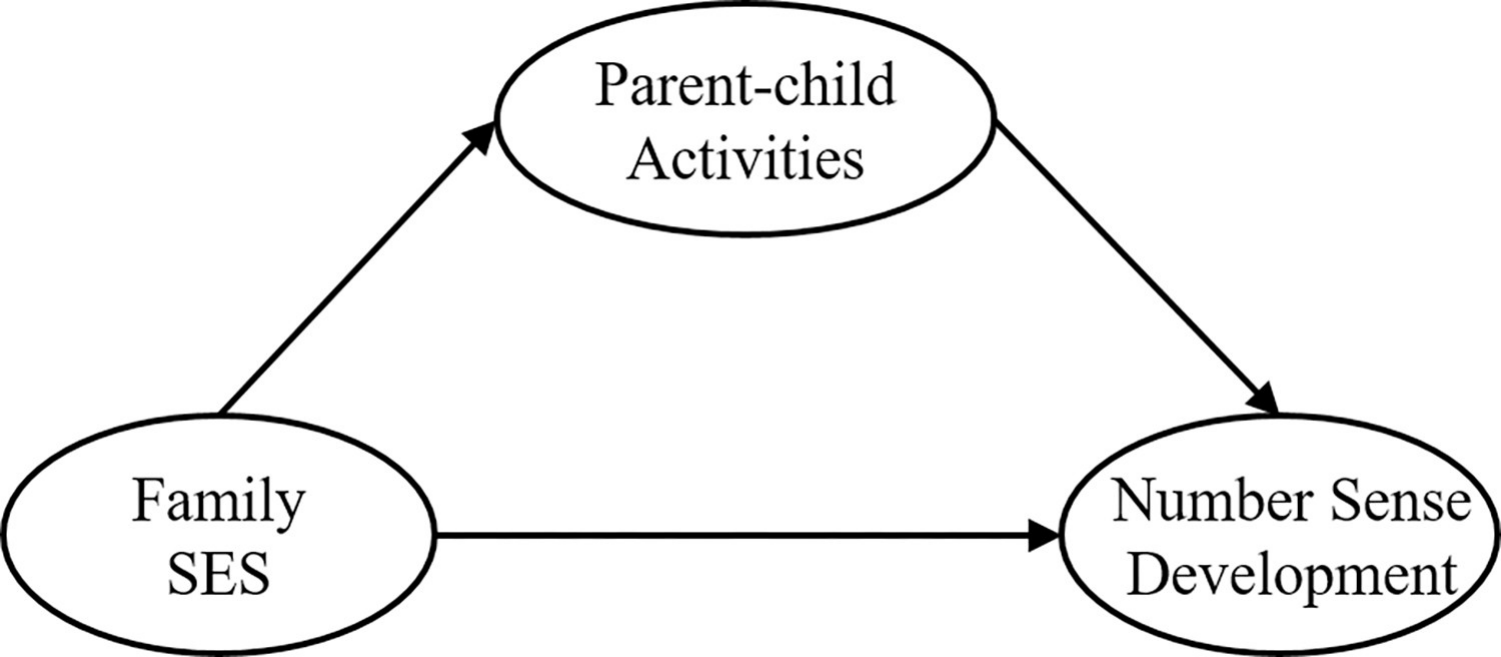
Hypothetical model diagram.

The family stress model suggests that when family economic stress is high, family distress will distract parents’ attention, and parents with low SES will show less emotional and behavioral concern for their children, resulting in fewer good parent-child interactions [35, 36]. On the other hand, parents of families with higher SES have a better economic base and more knowledge and time to organize various kinds of parent-child activities that are conducive to children’s physical and mental development. Additionally, parent-child activities are better in terms of content, frequency, and quality than those of families with lower SES. From this, it can be speculated that families with low SES have fewer parent-child activities that can promote the young children’s number sense development, thus affecting the young children’s number sense development. That is, SES may affect the young children’s number sense development through the intermediary of parent-child activities. Based on the above, we propose the hypothesis that parent-child activities play a mediating role between SES and the young children’s number sense development (H2). The model is shown in Fig 1.

To summarize, this study focuses on preschool children and discusses the relationship among socioeconomic status (SES), parent-child activities, and the young children’s number sense development. To address this issue, we collected data from hundreds of families and children, and explored the relationships between variables by establishing structural equation models. This mediation model helps us understand the link between SES and young children’s number sense development, the mechanisms by which it occurs, and provides theoretical support for interventions to improve the quality of parent-child activities and young children’s number sense development.

## Research methods

### Research subjects

The study was approved by the Research Ethics Committee of the Ningde Normal University. During the period from February 2022 to June 2022, 314 young children were randomly selected as subjects from kindergartens in Fujian Province using a convenience sampling method. The kindergarten head explained the purpose and content of the study to the parents and read the informed consent form to them. Full-time kindergarten teachers, who were trained by the researchers, conducted one-on-one tests on the young children, and questionnaires were distributed to the parents of the participating young children. During the test, the teachers emphasized the principle of confidentiality to both the young children and parents, and explained that they could withdraw from the test at any time. The entire data collection process is anonymous. Finally, a total of 296 responses were collected, and 226 responses were valid, accounting for 76.35%. Among them, 109 were boys, accounting for 48.2%, and 117 were girls, accounting for 51.8%. The young children had an average monthly age of 70.42 ± 3.47.

### Research tools

#### Family SES questionnaire

A self-made questionnaire on family economic and social status was utilized, which consisted of five questions. These were: (1) What is your family’s monthly income? (2) What is the educational background of the father? (3) What is the educational background of the mother? (4) What is the father’s occupation? (5) What is the mother’s occupation? The questionnaire utilized a Likert 5-point scoring system, whereby a higher score indicated a higher social and economic status for the family. In this study, the α coefficient of the questionnaire was 0.86.

#### Parent-child activities questionnaire

A self-made parent-child activities questionnaire was used in this study. The questionnaire consisted of four questions, namely: (1) Do you or your family tell stories or read together with your children at home? (2) Do you or your family often take your children to the library to borrow or read books? (3) Do you or your family play math games, such as counting, addition, and subtraction, with your children at home? (4) Do you or your family play educational games, such as building blocks, puzzles, chess, and cards, with your children at home? The questionnaire utilized Likert’s 3-point scoring system, with 1 representing "never" and 3 representing "often." A higher score indicates greater involvement in parent-child activities. In this study, the α coefficient of the questionnaire was 0.72.

### 5~6-year-old children’s number sense development scale

The 5~6-year-old children’s number sense development scale, compiled by Jiang [37], has been adopted. The scale comprises five dimensions: counting, number knowledge, number conversion, number grouping, and quantity estimation, with a total of 34 questions. It has been found to have good reliability and validity. The scale is scored on a binary scale, with 1 representing a correct answer and 0 representing an incorrect answer. The higher the score, the more developed the child’s number sense. In this study, the α coefficient of each dimension of the scale ranges from 0.66 to 0.81, and the α coefficient of the scale as a whole is 0.89.

### Statistical analysis

The study employed SPSS20 for descriptive and correlation analyses of the variables. In addition, Mplus7.4 was utilized to test the mediation model of parent-child activities between family SES and young children’s number sense developmental level. The MLR method was applied for parameter estimation, and the mediation effect was assessed using bias-corrected nonparametric percentile Bootstrap. This method can accurately estimate confidence intervals for the mediation effect of the product of regression coefficients and is suitable for non-normal data. Significance of the mediation effect was determined if the obtained effect value’s 95% confidence interval did not contain zero [38].

## Result

### Test for common method bias

To examine whether a significant common method bias existed in this study, an unrotated exploratory factor analysis was conducted on all variables. The results revealed three factors with characteristic roots greater than one. However, the variance explained by the first factor was only 29.58%, which is lower than the critical value of 40% [39]. Thus, the findings suggest that there was no significant common method bias in this study.

### Descriptive statistics for each variable

In order to test for a close relationship between variables and prepare for the establishment of a structural equation model, Pearson correlation analysis was used to analyze the relationship between variables. The results of the Pearson correlation analysis are shown in Table 1.

**Table 1:** Descriptive statistics of each variable and its correlation analysis results.

Variable	1	2	3	4	5	6
1 Gender^a^	—					
2 Month Age	0.10	—				
3 Kindergarten level ^b^	-0.01	-0.10	—			
4 family SES	-0.01	0.06	-0.11	—		
5 Parent-child activities	0.16*	0.07	-0.02	0.39***	—	
6 Number sense	0.12	0.10	0.18**	0.26***	0.27***	—
*M*				3.46	1.87	0.72
*SD*				0.97	0.34	0.20

Note: *n* = 416; a: Gender is a dummy-coded variable, where 0 = female and 1 = male; b: Kindergarten level is an ordinal variable, where 1 = provincial demonstration kindergarten, 2 = municipal/district demonstration kindergarten, and 3 = regular kindergarten; * *p* < 0.05, ** *p* < 0.01, *** *p* < 0.001.

Table 1 reveals that gender has a significant positive correlation with parent-child activities but has no significant correlation with family SES and the development level of number sense. The kindergarten level is positively correlated with the development level of number sense, but has no significant correlation with family SES and parent-child activities.

The main variables show a significant positive correlation. The Pearson correlation results indicate that the main variables are closely related, and thus, a structural equation model can be established for further analysis. Moreover, because gender and kindergarten level are significantly related to the research variables, they will be included as control variables in the model.

### Mediating role of parent-child activities

In order to test the hypotheses proposed in this study, structural equation models were developed to examine the mediating role of parent-child activities between young children’s family SES and levels of number sense, using each questionnaire topic/dimension as an observational indicator.

**Fig 2:**
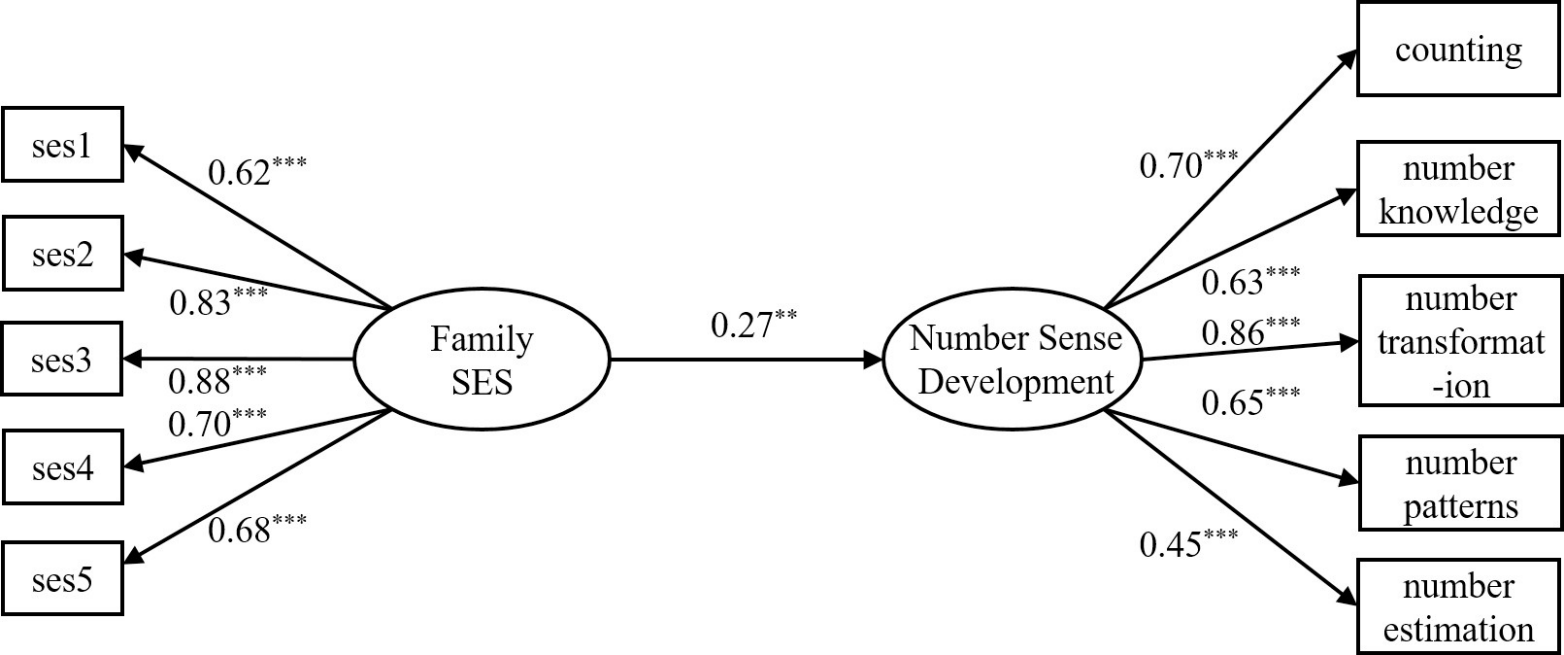
Direct effect model. Note: The numbers listed in the figure are standardized solutions; The level of the kindergarten is included as a control variable in the model, but is not shown in the figure for the sake of simplicity; ** *p* < 0.01, *** *p* < 0.001.

Firstly, the predictive effect of family SES on the level of number sense was tested. The results indicated that the overall model was well-fitted: *χ*^2^/*df* = 2.05, RMSEA = 0.07, CFI = 0.94, TLI = 0.93, SRMR = 0.06. Fig 2 displays the direct effect model, and it demonstrates that family SES significantly and positively predicts the level of number sense (*β* = 0.27, *p* < 0.01), thus validating H1. This finding suggests a strong association between the two variables. This shows that there is a close relationship between the two, and intermediary variables can be added to further analyze the influence mechanism of family SES on the level of young children’s number sense.

**Fig 3:**
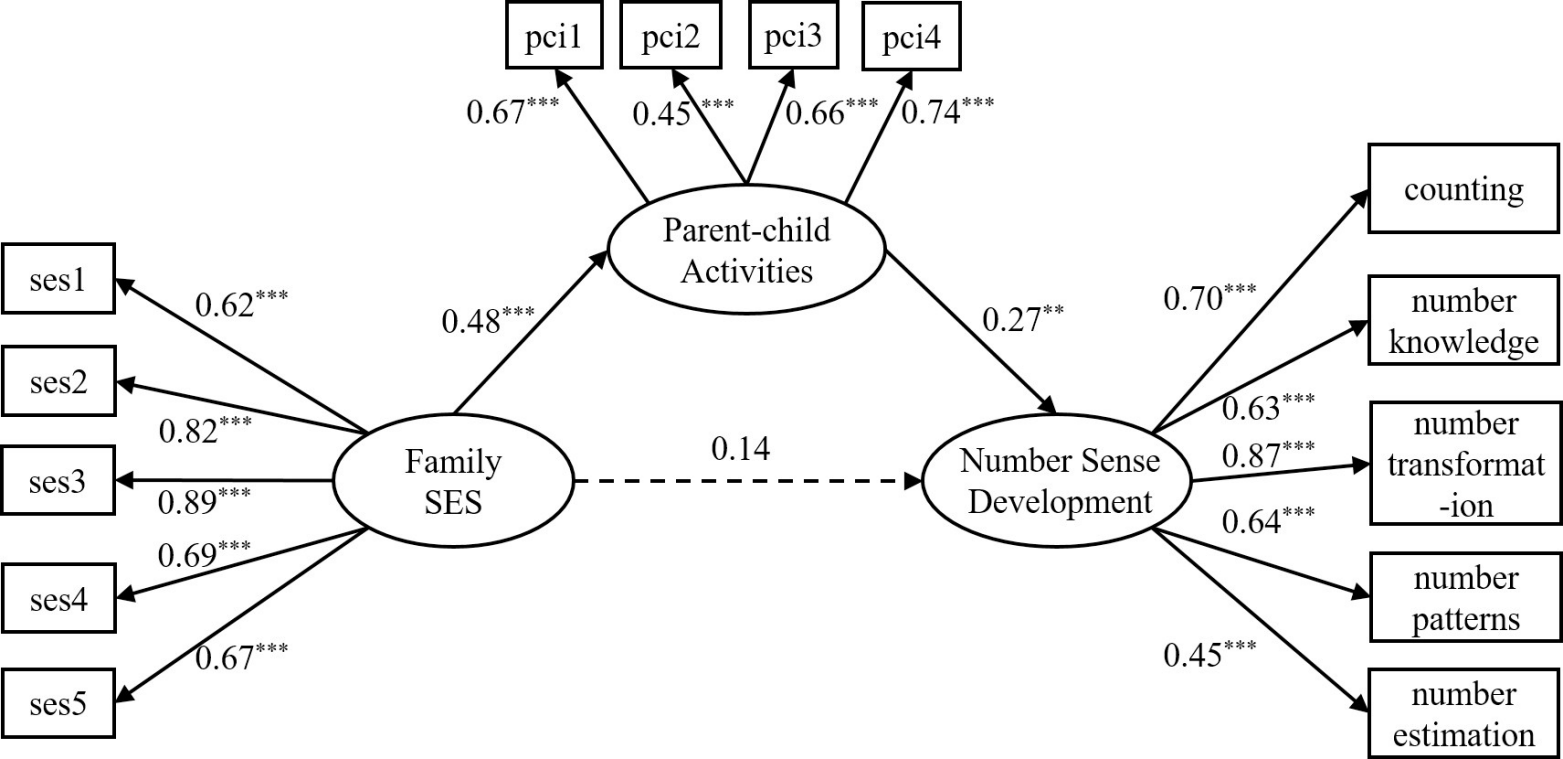
Mediation model diagram. Note: The numbers listed in the figure are standardized solutions; The level of the kindergarten and gender are included as control variable in the model, but is not shown in the figure for the sake of simplicity; "pci1-pci4" refers to the four items of parent-child activity; ** *p* < 0.01, *** *p* < 0.001.

Secondly, the study tested the mediating role of parent-child activities in the relationship between family SES and number sense in young children. The results indicated that the overall model fit well with *χ*^2^/*df =* 1.79, RMSEA = 0.06, CFI = 0.93, TLI = 0.91, SRMR = 0.06, and the mediation model is presented in Fig 3. The findings revealed that family SES significantly and positively predicted parent-child activity (*β* = 0.48, *p* < 0.001), and that parent-child activity significantly and positively predicted the level of numerical sense (*β* = 0.27, *p* < 0.01).

Finally, we tested the mediating effect of parent-child activities on the relationship between family SES and number sense by using bias-corrected nonparametric percentile bootstrap method with 5000 repeated samples. The results are presented in Table 2. The table shows that the mediating effect of parent-child activities between family SES and young children’s number sense is significant. The mediating effect value is 0.13, *p* < 0.01, 95%CI = [0.05, 0.25], and the mediating effect accounts for 48.52%, confirming hypothesis H2.

**Table 2:** Mediating effect value and Bootstrap test results.

Paths	Effect	Bootstrap confidence intervals (95%)
Total effect	0.27**	[0.11, 0.42]
Family SES→Parent-child Activities→Number sense	0.13**	[0.05, 0.25]
Direct effects	0.14	[-0.07, 0.33]

Note: ** *p* < 0.01.

## Discussion

Based on previous studies and the family stress model, this study constructs a mediation model to explore the relationship and the mechanism of influence between family SES and the young children’s number sense development in China. We collected data through a questionnaire and explored the relationship between family SES and number sense development, as well as the intermediary role of parent-child activities, through correlation analysis and a structural equation model. Our main findings are as follows: The correlation analysis showed a close relationship among family SES, parent-child activities, and the young children’s number sense development. The results of the mediation model revealed that family SES can positively predict the young children’s number sense development, and parent-child activities play an intermediary role in this relationship. This means that for young children with low family SES, increasing parent-child activities can reduce the influence of external unfavorable factors on their number sense development. The results of this study provide a reference for educators to formulate and promote the development of mathematics skills among children from low-SES families, which has important practical significance.

### Family SES can predict the young children’s number sense development

This study found that family SES had a significant predictive effect on young children’s number sense development, and after controlling for gender and kindergarten level. Young children’s number sense development is better in families with high SES. This result is consistent with previous studies [11, 15].

Compared with young children from lower family SES backgrounds, those from higher family SES backgrounds may be exposed to higher quality math activities and gain more math learning experience [40]. This is because families with high family SES can often provide young children with more educational resources and social support that are conducive to the development of young children. For example, they may play math games with young children more often and provide more opportunities for young children to participate in math thinking development classes.

However, the lack of educational resources and experience in mathematics in families with low family SES can put young children in these families at risk for developmental problems. Their development in counting, number relations, and number operation ability may be delayed before entering school. It is evident that the family’s input and support play an important role in the young children’s number sense development.

### The mediating role of parent-child activities

The results of this study show that parent-child activities play a partially mediating role in the influence of family SES on children’s number sense development. Firstly, SES may have a positive impact on parent-child activities, which is consistent with previous research results [41, 42], also confirming the views of the family investment model and the family stress model. Compared to parents with low SES, who are forced to focus solely on immediate needs, parents with higher SES will prioritize investing in their children’s human capital. They will invest more material and time in their children’s early childhood, thereby promoting early development and later educational success.

Specifically, families with high SES experience less pressure from economic difficulties and have higher family incomes. Therefore, they have the financial means to enroll their children in higher-quality preschool education programs and more time to engage in parent-child interaction activities. Studies have found that for every 10% increase in family income, parents’ investment in early childhood time increases by 0.9 standard deviations [43]. At the same time, parents with high socioeconomic status have a higher education level. Parents with a high education level can realize the importance of the early parenting environment for children’s development, and these parents are willing to spend more time interacting with children [44, 45]. Conversely, lower levels of education may limit parents’ awareness of the need to provide a nurturing environment for their children, and therefore, positive parenting activities are less likely to occur.

Secondly, family parent-child activities may positively affect the development level of children’s number sense, which is consistent with previous research conclusions [26, 46]. In parent-child activities, the active interaction between parents and children not only provides children with a rich learning environment with timely feedback but also helps children accumulate mathematical experience and acquire basic mathematical skills such as number recognition and counting. Additionally, this study found that the content related to cultivating reading ability, spatial ability, and gaming ability in parent-child activities is conducive to the young children’s number sense development. This finding expands upon previous research. Previous studies have individually confirmed that activities such as parent-child reading and building blocks games each affect the development of children’s mathematical abilities [26, 46]. This study further discovered that various types of colorful daily parent-child activities within the family can collectively contribute to the children’s number sense development.

Studies have pointed out that reading skills and mathematics are related [47, 48]. Having good reading ability can effectively help children understand mathematical story questions [49–51]. Puzzle games such as building blocks and chess can not only make children gain more cognitive experience in parent-child interaction but also help to improve their thinking logic, thus promoting the young children’s number sense development.

### Education suggestions

#### Fully utilize the compensatory function of kindergarten education to mitigate the adverse effects of low SES within families on the young children’s number sense development

Young children from low SES families have limited access to educational resources at home, which can limit their learning opportunities. However, improving a family’s SES often requires macro-social reform and the long-term efforts of multiple generations, making it a complex and long-term project. However, external forces such as kindergarten education can play a compensatory role, making it more feasible and effective to provide additional resources to support these young children.

On one hand, kindergartens can communicate the importance of parent-child activities to parents through parent-child meetings, demonstrate how to engage in such activities during open days, encourage parents to visit the library with their children, and play games together. Thus guiding parents in conducting meaningful parent-child interactions. On the other hand, kindergarten teachers can establish shared learning spaces and provide more books and learning opportunities for these young children, helping to alleviate the negative impact of their unfavorable family situations.

#### Parents should engage in diversified parent-child activities as much as possible and fully utilize the role of these activities in promoting the young children’s number sense development

According to Bourdieu’s theory, the cultural capital owned by families plays a stronger role in class definition and class barriers than economic and social capital [28, 52]. As an important part of the cultural capital experienced by young children, parent-child activities can effectively promote the young children’s number sense development. "Life course theory" emphasizes that the younger a child is, the greater the influence of family environmental factors [53]. To prevent young children from low SES families from falling behind at the starting point of life due to the lack of family resources, low SES families should pay more attention to the power of parent-child activities and provide more opportunities for young children to engage with diverse learning stimuli through various parent-child activities. This will not only aid in young children’s mathematics learning but also compensate for their parents’ educational level weaknesses and their own SES in the family, and enhance the young children’s number sense development.

Therefore, parents should always accompany their children to engage in some parent-child activities at home, and these activities are not limited to specialized mathematics activities. Reading activities and game activities can also be beneficial. It is not necessary to think that they cannot teach their children mathematics because of their low academic qualifications. Reading with children, taking them to the library, playing with building blocks, and playing chess with them can also contribute to promoting the young children’s number sense development.

### Research limitations and outlook

There are some limitations to this study: (1) The study only includes research subjects from one province. To increase the ecological validity of the study, future research can include subjects from other provinces; (2) The research design is cross-sectional, which cannot establish a causal relationship among variables. In the future, a longitudinal research design can be adopted to reveal the causal relationship between independent and dependent variables.

## Conclusion

In this study, a mediation model was constructed to investigate the relationship between family SES, parent-child activities, and the young children’s number sense development. The following conclusions were drawn: (1) family SES can significantly and positively predict the young children’s number sense development, and (2) Parent-child activities play an intermediary role in the relationship between family SES and the young children’s number sense development.

## References

[1] National council of teachers of mathematics. Principles and standards for school mathematics. Reston, VA: National Council of Teachers of Mathematics, 2000; 32.

[2] Wen-bin X, Ping Y. Number Sense and Its Development. Journal of mathematics education. 2007(02):8–11. 10.3969/j.issn.1004-9894.2007.02.003

[3] Beswick K. Why Does 0.999… = 1?: A Perennial Question and Number Sense. Australian Mathematics Teacher, The, 2004; 60(4): 7–9. 10.3316/INFORMIT.185503582869670

[4] Leibovich T, Katzin N, Harel M, Henik A. From“sense of number”to“sense of magnitude”: The role of continuous magnitudes in numerical cognition. Behav Brain Sci. 2017; 40: e164. doi: 10.1017/S0140525X16000960 27530053

[5] Jordan N. C., Glutting J., & Ramineni C. The importance of number sense to mathematics achievement in first and third grades. Learning and Individual Differences,2010; 20(2): 82–88. doi: 10.1016/j.lindif.2009.07.004 20401327 PMC2855153

[6] Locuniak M. N., & Jordan N. C. Using kindergarten number sense to predict calculation fluency in second grade. Journal of Learning Disabilities, 2008; 41(5): 451–459. doi: 10.1177/0022219408321126 18768776 PMC3935894

[7] Sirin S. R. Socioeconomic status and academic achievement: A meta-analytic review of research. Review of Educational Research, 2005; 75(3): 417–453. 10.3102/00346543075003417

[8] Bae D., & Wickrama K. A. S. Family socioeconomic status and academic achievement among korean adolescents: Linking mechanisms of family processes and adolescents’ time use. The Journal of Early Adolescence, 2015; 35(7): 1014–1038. 10.1177/0272431614549627

[9] Dowker A. Early Identification and Intervention for Students with Mathematics Difficulties. Journal of Learning Disabilities, 2005; 38(4): 324–332. doi: 10.1177/00222194050380040801 16122064

[10] Chard D. J., Baker S.K., Clarke B., Jung Johann K., Davis K., & Smolkowski K. Preventing early mathematics difficulties: The feasibility of a rigorous kindergarten mathematics curriculum. Learning Disability Quarterly, 2008; 31(1): 11–20. 10.2307/30035522

[11] Jordan N. C, Kaplan D, Locuniak M. N, Ramineni C. Predicting first‐grade math achievement from developmental number sense trajectories. Learn Disabil Res Pr. 2007; 22(1): 36–46. 10.1111/j.1540-5826.2007.00229.x

[12] Jordan N.C., Kaplan D., Ramineni C., &Locuniak M.N. Development of number combination skill in the early school years: When do fingers help? Developmental Science, 2008; 11(5): 662–668. doi: 10.1111/j.1467-7687.2008.00715.x 18801121

[13] Ramey C. T., & Ramey S.L. Early childhood education: The journey from efficacy research to effective practice.Handbook of research methods in developmental science, 2005; 233–248. 10.1002/9780470756676.ch12

[14] Duncán G.J., & Brooks-Gunn J. (2001). Poverty, welfare reform, and children’s achievement. In Biddle (Ed.), Social class, poverty, and education: Policy and practice (pp.49–76). New York:Routledge Almer.

[15] Klibanoff R. S., Levine S. C., Huttenlocher J., Vasilyeva M., & Hedges L. V. (2006). Preschool children’s mathematical knowledge: The effect of teacher "math talk." Developmental Psychology,2006; 42(1): 59–69. doi: 10.1037/0012-1649.42.1.59 16420118

[16] Hao Y. Study on the Social and Economic Status of Students’ Families and Its Relationship with Mathematics Academic Achievements. In: Editor, editor Academic Annual Meeting of Professional Committee of Basic Education Evaluation of China Education Society; 2012; Beijing, China. 2012.

[17] Chen Y, Yang X. The Effects of Family Socioeconomic Status on Mathematical Achievements: The Chain Mediation Model of Parent-Child Communication and Academic Self Efficacy. Chinese Journal of Applied Psychology. 2020;26(01):66–74.

[18] Bronfenbrenner U. Ecology of the family as a context for human development: Research perspectives. Developmental Psychology, 1986, 22(6): 723–742. 10.1037/0012-1649.22.6.723

[19] Tramonte L. & Willms J.D. Cultural capital and its effects on education outcomes. Economics of Education Review, 29(2), 200–213. Elsevier Ltd. Retrieved February 14, 2024 from https://www.learntechlib.org/p/206835/.

[20] Social experience in the development of representation thought: Distancing theory. In Sigle I. E, Brodzinsky D. M, & Golinkoff R. M (Eds.). New directions in Piagetian theory and practice. Hillsdale, NJ: Erlbaum, 216

[21] Anderson A. (1997). Families and mathematics: A study of parent-child interactions. Journal for Research in Mathematics Education, 1997; 28(4): 484–511. 10.2307/749684

[22] Conger R. D., Conger K. J., & Martin M. J. Socioeconomic status, family processes, and individual development. Journal of Marriage and Family, 2010; 72(3): 685–704. doi: 10.1111/j.1741-3737.2010.00725.x 20676350 PMC2910915

[23] Tazouti Y., & Jarlégan A. The mediating effects of parental self-efficacy and parental involvement on the link between family socioeconomic status and children’s academic achievement. Journal of Family Studies,2019; 25(3): 250–266. 10.1080/13229400.2016.1241185

[24] Liu C. L. How parents can help children with their achievement in mathematics. EduMath, 2001; 13: 12–24.

[25] LeFevre J.-A., Skwarchuk S.-L., Smith-Chant B. L., Fast L., Kamawar D., & Bisanz J. Home numeracy experiences and children’s math performance in the early school years. Canadian Journal of Behavioural Science / Revue canadienne des sciences du comportement, 2009; 41(2): 55–66. 10.1037/a0014532

[26] Huang J., Chen Q. A Study of Family Cultural Capital and Mathematics Learning Children’s. Journal of Psychological Science, 2011; 34(6):1385–1389.

[27] Ribner A, Silver AM, Elliott L, Libertus ME. Exploring effects of an early math intervention: The importance of parent-child interaction. Child Development, 2022; 94(2): doi: 10.1111/cdev.13867 36321367 PMC9991950

[28] Pan Y, Liu S, Yang Q. The Relationship Between Math Learning Activities at Home and Young Children’s Number Sense: A Moderated Mediation Model. Chinese Journal of Special Education. 2019; (04):75–82. 10.3969/j.issn.1007-3728.2019.04.012

[29] Carey S. Bootstrapping & the Origin of Concepts. Daedalus, 2004; 133(1): 59–68. http://www.jstor.org/stable/20027897

[30] Gordon P. Numerical Cognition without Words: Evidence from Amazonia. Science, 2004; 306(5695): 496–499. doi: 10.1126/science.1094492 15319490

[31] Gelman R., & Butterworth B. Number and language: how are they related?. Trends in cognitive sciences, 2005; 9(1): 6–10. doi: 10.1016/j.tics.2004.11.004 15639434

[32] Passolunghi M. C., Cargnelutti E., & Pastore M. The contribution of general cognitive abilities and approximate number system to early mathematics. The British journal of educational psychology, 2014; 84(4): 631–649. doi: 10.1111/bjep.12054 25175790

[33] Wei W., Yuan H., Chen C., & Zhou X. Cognitive correlates of performance in advanced mathematics. The British journal of educational psychology, 2012; 82(1): 157–181. doi: 10.1111/j.2044-8279.2011.02049.x 22429063

[34] Mix K. S., Levine S. C., Cheng Y. L., Young C., Hambrick D. Z., Ping R., et al. Separate but correlated: The latent structure of space and mathematics across development. Journal of experimental psychology. General, 2016; 145(9): 1206–1227. doi: 10.1037/xge0000182 27560854

[35] Bradley R. H., & Corwyn R. F. Socioeconomic status and child development. Annual review of psychology, 2002; 53: 371–399. doi: 10.1146/annurev.psych.53.100901.135233 11752490

[36] Conger R. D., Wallace L. E., Sun Y., Simons R. L., McLoyd V. C., & Brody G. H. Economic pressure in African American families: a replication and extension of the family stress model. Developmental psychology, 2002; 38(2): 179–193. 11881755

[37] Jiang Y. Development and Application of Number Sense Test for 5–6 Years Old Children: National Kaohsiung Normal University; 2022.

[38] Wen Z, Ye B. Analyses of mediating effects: the development of methods and models. Advances in psychological Science. 2014;22(5):731–45.

[39] Tang D, Wen Z. Statistical approaches for testing common method bias: Problems and suggestions. Journal of Psychological Science. 2020(1):215–23. 10.16719/j.cnki.1671-6981.20200130

[40] Elliott L. and Bachman H.J. Parents Educational Beliefs and Children’s Early Academics: Examining the Role of SES. Children and Youth Services Review, 2018; 91: 11–21. 10.1016/j.childyouth.2018.05.022

[41] Bradley R. H., Corwyn R. F., McAdoo H. P., & Coll C. G. The home environments of children in the United States part I: variations by age, ethnicity, and poverty status. Child development, 2001; 72(6): 1844–1867. doi: 10.1111/1467-8624.t01-1-00382 11768149

[42] Larson K., Russ S. A., Nelson B. B., Olson L. M., & Halfon N. Cognitive ability at kindergarten entry and socioeconomic status. Pediatrics, 2015; 135(2): e440–e448. doi: 10.1542/peds.2014-0434 25601983

[43] Carneiro P. and Ginja R. Partial Insurance and Investments in Children. Partial Insurance and Investments in Children, The Economic Journal, 2016; 126(596): F66–F95. 10.1111/ecoj.12421

[44] Sohr-Preston S. L., Scaramella L. V., Martin M. J., Neppl T. K., Ontai L., & Conger R. Parental socioeconomic status, communication, and children’s vocabulary development: a third-generation test of the family investment model. Child development, 2013; 84(3): 1046–1062. doi: 10.1111/cdev.12023 23199236 PMC3594401

[45] Roubinov D. S., & Boyce W. T. Parenting and SES: relative values or enduring principles?. Current opinion in psychology, 15, 2017; 162–167. doi: 10.1016/j.copsyc.2017.03.001 28503655 PMC5423399

[46] Berkowitz T, Schaeffer M W, Maloney E A, et al. Math at home adds up to achievement in school. Science, 2016; 351(6278): 1161. https://doi.org/1126/science.aac7427 10.1126/science.aad8555 26965620

[47] Cirino P. T., Child A. E., & Macdonald K. T. Longitudinal predictors of the overlap between reading and math skills. Contemporary Educational Psychology, 2018; 54: 99–111. doi: 10.1016/j.cedpsych.2018.06.002 30559576 PMC6294126

[48] Koponen T., Eklund K., Heikkilä R., Salminen J., Fuchs L., Fuchs D., et al. Cognitive correlates of the covariance in reading and arithmetic fluency: Importance of serial retrieval fluency. Child Development, 2020; 91(4): 1063–1080. doi: 10.1111/cdev.13287 31292957

[49] Lewis C., Hitch G. J., & Walker P. The prevalence of specific arithmetic difficulties and specific reading difficulties in 9- to 10-year old boys and girls. Child Psychology & Psychiatry & Allied Disciplines, 1994; 35(2): 283–292. doi: 10.1111/j.1469-7610.1994.tb01162.x 8188799

[50] Landerl K., & Moll K. Comorbidity of learning disorders: prevalence and familial transmission. Journal of child psychology and psychiatry, and allied disciplines, 2010; 51(3); 287–294. doi: 10.1111/j.1469-7610.2009.02164.x 19788550

[51] Moll K., Snowling M. J., Göbel S. M., & Hulme C. Early language and executive skills predict variations in number and arithmetic skills in children at family-risk of dyslexia and typically developing controls. Learning and Instruction, 2015; 38: 53–62. doi: 10.1016/j.learninstruc.2015.03.004 26412946 PMC4567032

[52] Sun Y L. The Influence on Students Growth Caused by the Family Cultures. Journal of Educational Science of Hunan Normal University, 2006; 04: 44–46.

[53] Lucas SR. Selective attrition in a newly hostile regime: The case of 1980 sophomores. Soc Forces. 1996;75(2):511–33. 10.2307/2580411

